# Does Nucleos(t)ide Analogues Treatment Affect Renal Function in Chronic Hepatitis B Patients Who Have Already Decreased eGFR? A Longitudinal Study

**DOI:** 10.1371/journal.pone.0149761

**Published:** 2016-03-10

**Authors:** Ming-Chao Tsai, Chien-Hung Chen, Po-Lin Tseng, Chao-Hung Hung, King-Wah Chiu, Kuo-Chin Chang, Yi-Hao Yen, Ming-Tsung Lin, Tsung-Hui Hu

**Affiliations:** 1 Division of Hepato-Gastroenterology, Department of Internal Medicine, Chang Gung Memorial Hospital-Kaohsiung Medical Center, Chang Gung University College of Medicine, Kaohsiung, Taiwan; 2 Graduate Institute of Clinical Medical Sciences, Chang Gung University College of Medicine, Kaohsiung, Taiwan; Yonsei University College of Medicine, REPUBLIC OF KOREA

## Abstract

This study aimed to assess the renal function in chronic hepatitis B (CHB) patients who received nucleos(t)ide analogues (NAs) therapy using estimated glomerular filtration rate (eGFR) titer. We performed a longitudinal observational study of 37 tenofovir-, 42 telbivudine-, and 62 entecavir-naïve CHB patients, who had impaired renal function (eGFR, 90–30 ml/min/1.73m^2^) without history of diabetes, hypertension, and chemotherapy. Calculation and evaluation of eGFR was performed with the Modification of Diet in Renal Disease, Chronic Kidney Disease Epidemiology Collaboration, and Cockcroft-Gault formula at pretreatment, at baseline, and after the 1^st^ and 2^nd^ year of treatment. The eGFR was significantly increased in patients given telbivudine or entecavir (*p* = 0.003 and *p* = 0.012, respectively), but the eGFR was decreased in patients given tenofovir (*p* = 0.001) after 2 years of treatment. Of all patients, eGFR was stable one year prior to treatment. If we analyzed the renal function by change of chronic kidney disease (CKD) category with a change of 25% of eGFR, the proportion of uncertain drop (drop in CKD category with <25% decrease in eGFR) and certain drop (drop in CKD category with ≧25% decrease in eGFR) in tenofovir group was smaller (5.4%) than those of telbivudine (12.9%) or entecavir (6.5%). Furthermore, telbivudine had the lowest stable rate (76.2%), the highest certain rise rate (9.5%), and certain drop rate (7.1%) compared to the other groups (*p* = 0.049). In conclusion, in NAs-naïve CHB patients with impaired renal function, telbivudine and entecavir resulted in a significant increase in eGFR while tenofovir resulted in a significant decrease after a 2-year treatment. Interestingly, TDF had the lowest proportion of patients reclassified to certain and uncertain drop groups; in contrast, LdT had a higher proportion in both raise and drop groups. The outcomes of this renal effect remain to be determined.

## Introduction

Chronic hepatitis B (CHB) is one of the most common infectious diseases, affecting more than 350 million people worldwide [1]. Over the last 15 years, the outcome of CHB has dramatically improved due to the advent of effective antiviral agents [2]. To date, five nucleos(t)ide analogs (NAs) are approved for the treatment of hepatitis B. Of these NAs, entecavir (ETV) and tenofovir disoproxil fumarate (TDF) are the preferred first-line agents due to their high genetic barrier and virological remission. Although NAs are effective in suppressing the virus, the treatment duration is not well-defined and most patients require long-term treatment. Therefore, patient safety is also an issue.

All NAs approved for HBV predominantly undergo renal clearance and harbor dose-dependent kidney toxicity by various mechanisms, including alterations in renal tubular transporters, apoptosis, and mitochondrial toxicity [3]. Hence, the change of renal function is another major issue in CHB patients under NAs treatment. Most recently, numerous prospective and real-world studies indicated an improvement of creatinine clearance in several patient subgroups with telbivudine (LdT) therapy via an unknown mechanism [4–8]. In contrast to LdT, TDF-associated renal dysfunction has been described in several cases and studies in HIV-infected patients [9, 10]. Nevertheless, studies in phase III trial and real-life have widely reported that TDF is not an independent predictor for significant deterioration of renal function [11, 12]. Although these studies used eGFR instead of creatinine to evaluate renal function, it is difficult to draw any conclusions regarding the potential nephrotoxic or nephroprotective effect of a given drug due to multiple factors affecting renal function. There were still many limitations in each study, including the lack of detailed drug history except NAs, the severity of diabetes and/or hypertension, and the eGFR status prior to NAs treatment, which might all affect renal function. In order to overcome these limitations, we must identify a group of CHB patients with the least amount of factors affecting eGFR and analyze eGFR in a longitudinal study before and after treatment to closely monitor the effect of NAs in CHB patients. In this study, we retrospectively designed a group of CHB patients with impaired renal function, no prior history of diabetes and hypertension, and no diuretics treatment when starting NAs treatment. The serial eGFR was analyzed from one year prior to treatment to two years after treatment.

## Material and Methods

### Study design and patient population

We performed a retrospective-prospective cohort study using data from Chang Gung Memorial Hospital, Kaohsiung Medical Center, Taiwan. This study protocol had previously been approved by the ethical committees of Chang Gung Memorial Hospital with signed informed consent from all patients. Through a computerized database, first we identified all CHB patients who were treated with either TDF, LdT or ETV between June 2008 and June 2013, and then manually reviewed their medical records to determine eligibility. The inclusion criteria were treatment with at least 2-year NAs and pre-existing renal impairment [eGFR between 30 and 90 mL/min/1.73m^2^, eGFR was calculated using the Modification of Diet in Renal Disease (MDRD)]. Those who met any of the following criteria were excluded: history of diabetes, hypertension, any malignant disease underwent chemotherapy, organ transplantation, superimposed infection with hepatitis C virus or HIV, and no serum creatinine data before and after NAs treatment.

### Assessment of renal function

Assessment of renal function was based on eGFR using the Modification of Diet in Renal Disease (MDRD) [13], the Chronic Kidney Disease Epidemiology Collaboration (CKD-EPI) [14], and Cockcroft-Gault [15] at pretreatment (one year prior to treatment), at baseline (initiation of treatment), and after the 1^st^ and 2^nd^ year of treatment. Serum creatinine data was recorded from outpatient department, and values from patients with sepsis or gastrointestinal bleeding were excluded from analysis.

Furthermore, we defined the change in kidney function as a certain rise (rise in CKD category with ≧25% increase in eGFR), an uncertain rise (rise in CKD category with <25% rise in eGFR), stable (no change in CKD category), an uncertain drop (drop in CKD category with <25% decrease in eGFR), and a certain drop (drop in CKD category with ≧25% decrease in eGFR). The change in eGFR was calculated by [2^nd^ year eGFR-baseline eGFR]/baseline eGFR x 100. Categories of chronic kidney disease (CKD) were defined based on eGFR: ≧90, 60–89, 59–30, and <30 ml/min/1.73m^2^, respectively.

### Statistical analyses

The differences in continuous and categorical variables across the three groups were assessed using ANOVA and Chi-square, as appropriate. The change in eGFR among pretreatment, baseline and after one or two years was analyzed using the paired *t*-test for each group. All statistical analyses were performed using the SPSS software version 17.0 (SPSS Inc., Chicago, IL).

## Results

### Baseline characteristics of the study population

**Fig 1:**
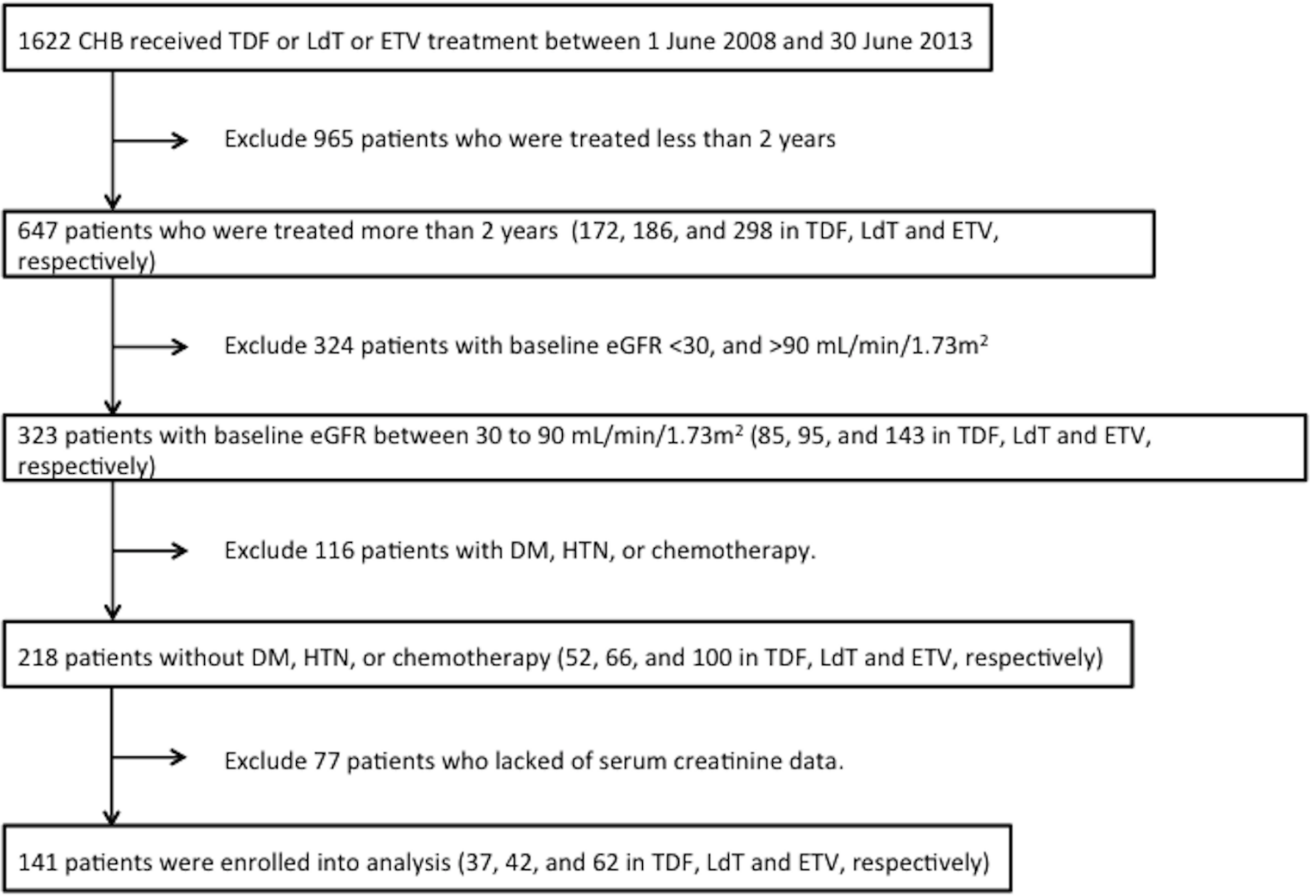
Schematic flowchart of the enrollment process.

Out of 1622 consecutive patients screened, a total of 141 CHB patients receiving TDF (n = 37), LdT (n = 42) or ETV (n = 62) for at least 2 years without history of diabetes, hypertension and chemotherapy, and with a baseline eGFR between 30 and 90 ml/min/1.73m^2^ was enrolled into the analysis (Fig 1). The baseline characteristics and confounding drugs are shown in Table 1 (and online S1 Dataset). Overall, 77%, 55%, and 16% of subjects were male, liver cirrhotic, and had HCC, respectively. Three groups were matched in terms of factors already known to influence renal function, such as gender, age, liver cirrhosis, hepatocellular carcinoma, and pre-existing renal function including level of creatinine and eGFR. All cirrhotic cases were in Child Pugh A without ascites and diuretics prescription. Only the mean HBV DNA was lower in the LdT group than in the ETV and TDF groups.

**Table 1:** Baseline characteristics of the study population.

	Total (n = 141)	Tenofovir (n = 37)	Telbivudine (n = 42)	Entecavir (n = 62)	*p*-value
Age (years)	55.2 ± 12.2	53.6 ± 12.6	56.6 ± 12.9	55.2 ± 11.5	0.561
Male gender, n (%)	108 (77%)	32 (86%)	30 (71%)	46 (74%)	0.505
ALT (U/L)	200 ± 337	262 ± 435	146 ± 245	200 ± 323	0.315
Platelet (10^9^/L)	150 ± 54.7	164.5 ± 41.6	153.7 ± 61.8	149.6 ± 56.5	0.501
HBeAg, n (%)	37 (26%)	10 (27%)	9 (21%)	18 (29%)	0.683
HBV-DNA (log_10_ copies/ml)	6.3 ± 1.3	6.3 ± 1.3^a^	6.0 ± 1.4^a^^,^^b^	6.4 ± 1.2^b^	0.039
Liver cirrhosis, n (%)	74 (52%)	15 (41%)	23 (55%)	36 (58%)	0.226
HCC, n (%)	22 (16%)	4 (11%)	9 (21%)	9 (15%)	0.410
Creatinine (mg/dl)	1.0 ± 0.2	0.9 ± 0.2	1.0 ± 0.2	1.0 ± 0.2	0.782
Estimated GFR (ml/min/1.73m^2^)					
by MDRD	75.7 ± 10.5	78.3± 11.6	73.5 ± 10.8	75.6 ± 9.5	0.125
by CKD-EPI	82.4 ± 13.1	85.6 ± 14.7	79.3 ± 12.9	82.6 ± 11.9	0.106
by CG	78.0 ± 15.4	79.9 ± 16.3	75.5 ± 15.7	78.6 ± 14.8	0.422
Concomitant drugs^c^, n (%)					
NSAID	27 (19.1%)	6 (16.2%)	9 (21.4%)	12 (19.4%)	0.840
Diuretics	15 (10.6%)	3 (8.1%)	7 (16.7%)	5 (8.1%)	0.319
Statin	5 (3.5%)	2 (3.2%)	1 (12.4%)	2 (5.4%)	0.256
ACEI	3 (2.1%)	1 (1.6%)	1 (2.4%)	1 (2.7%)	0.927
Cardiovascular drugs^d^	8 (5.7%)	2 (5.4%)	2 (4.8%)	4 (6.5%)	0.932

Data are expressed as mean±standard deviation or number (percentage).

^a^ Significant differences between tenofovir and telbivudine

^b^ Significant differences between entecavir and telbivudine with LSD post hoc correction or chi-squared test

^c^ All concomitant medications are represented as binary parameters

^d^ include isosorbide dinitrate, beta-blockers, and anticoagulants

Abbreviation: HBV, hepatitis B virus; HBeAg, hepatitis B e antigen; ALT, alanine aminotransferase; GFR, glomerular filtration rate; MDRD, modification of diet in renal disease; CKD-EPI, Chronic Kidney Disease Epidemiology Collaboration; CG, Cockcroft-Gault; ACEI, angiotensin-converting-enzyme inhibitor; NSAID, nonsteroidal anti-inflammatory drug

There were two, three, and six cases with eGFR less than 50 mL/min/1.73m^2^ at baseline (initiation of treatment), and after the 1^st^ and 2^nd^ year of treatment, respectively. The drug dosages were all tapered from q.d. to q.o.d.

### Telbivudine and Entecavir increase eGFR significantly, but Tenofovir decreases

**Fig 2:**
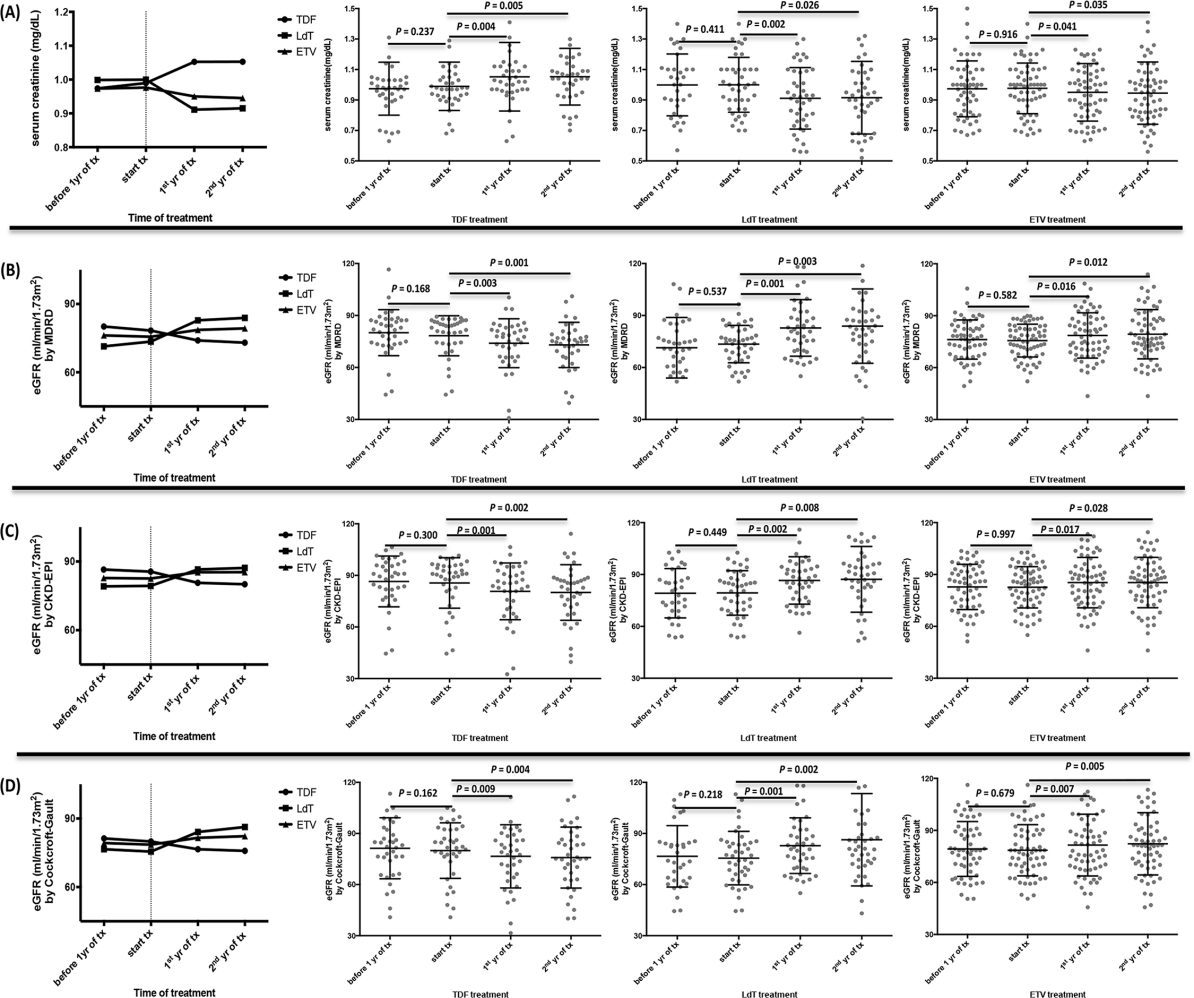
Changes of renal function-associated indicators among tenofovir, telbivudine and entecavir groups before and after treatment. (A) by serum creatinine (B) eGFR calculated by MDRD (C) eGFR calculated by CKD-EPI (D) eGFR calculated by Cockcroft-Gault. Horizontal bar indicates mean levels with standard deviation.

The median levels of serum creatinine and eGFR 1 year prior to treatment to two years after treatment are shown in Fig 2. There were no significant differences between one year prior to treatment and the start of treatment in terms of serum creatinine, eGFR by MDRD, CKD-EPI, and the Cockcroft-Gault equation. We only showed the data of eGFR by MDRD. (TDF: 80.1 ± 13.3 → 78.3 ± 11.6 ml/min/1.73m^2^, *p* = 0.168; LdT: 71.4 ± 17.5 → 73.5 ± 10.8 ml/min/1.73m^2^, *p* = 0.537; ETV: 76.2 ± 11.3 → 75.6 ± 9.5 ml/min/1.73m^2^, *p* = 0.582), but the eGFR significantly increased in patients receiving LdT (73.5 ± 10.8 → 83.9 ± 21.4 ml/min/1.73m^2^, *p* = 0.003) and ETV (75.6 ± 9.5 → 79.3 ± 14.2 ml/min/1.73m^2^, *p* = 0.012), and significantly decreased in TDF (78.3 ± 11.6 → 73.0 ± 13.1 ml/min/1.73m^2^, *p* = 0.001) after two years of treatment, as compared to baseline, which are all shown in using the serum creatinine (Fig 2A), eGFR by MDRD (Fig 2B), CKD-EPI (Fig 2C), and the Cockcroft-Gault equation (Fig 2D).

### Telbivudine causes great fluctuation in renal function

Further analysis by change in CKD category and at least a 25% change in eGFR showed that 123 patients (87.2%) had a stable kidney function (no change in CKD category), 4 (2.8%) had a certain rise, and 4 (2.8%) had a certain drop in kidney function (Table 2). Among these three groups, TDF had the smaller proportion of uncertain drop and certain drop (5.4%) than those of LdT (12.9%) or ETV (6.5%). LdT had the lowest stable rate (76.2%), the highest certain rise rate (9.5%), and certain drop rate (7.1%) (*p* = 0.049). Overall, seven patients expired during the follow-up period (mean, 55 months). Of these patients, one was in certain rise, one was in uncertain rise, four was in stable, and one was in certain drop groups. All cases were complicated by liver cirrhosis, such as HCC, spontaneous bacterial peritonitis or esophageal varices bleeding.

**Table 2:** A comparison of the change in renal function* between study groups.

	Tenofovir (n = 37)	Telbivudine (n = 42)	Entecavir (n = 62)	Total (n = 141)	Mortality^※^ (n = 7)
Certain rise	0	4 (9.5%)	0	4 (2.8%)	1 (25%)
Uncertain rise	1 (2.7%)	1 (2.4%)	1 (1.6%)	3 (2.1%)	1 (33.3%)
Stable	34 (91.9%)	32 (76.2%)	57 (91.9%)	123 (87.2%)	4 (3.3%)
Uncertain drop	1 (2.7%)	2 (4.8%)	4 (6.5%)	7 (5%)	0
Certain drop	1 (2.7%)	3 (7.1%)	0	4 (2.8%)	1 (25%)

*The groups for the change in kidney function were defined as: certain rise: rise in CKD category with ≧25% increase in eGFR; uncertain rise: rise in CKD category with <25% rise in eGFR; stable: no change in CKD category; uncertain drop: drop in CKD category with <25% decrease in eGFR; certain drop: drop in CKD category with ≧25% decrease in eGFR.

^※^All subjects died due to liver cirrhotic complications.

## Discussion

Our results indicated that after two years of treatment, LdT and ETV therapy is correlated with improved eGFR, while TDF therapy is associated with decreased eGFR in CHB patients with impaired renal function. All three groups revealed stable eGFR before treatment. To our knowledge, this is the first longitudinal study that compared the effects on approved NAs by eGFR from one year prior to treatment to two years after treatment.

Recently, Gane *et al*. [8] reported a comprehensive analysis of renal function in the LdT clinical trial database. This database indicated that eGFR increased by 14.9 mL/min/1.73m^2^ from baseline to year-4 (*p* < 0.0001). The improvement in renal function was more evident in patients with mildly reduced baseline eGFR (60–90 mL/min/1.73m^2^). In 2013, we published a retrospective match-control study comparing 230 CHB patients who had received 2 years of LdT or ETV that indicated significant eGFR improvement in both groups at year 2 [4]. Similarly, patients with impaired baseline eGFR (< 90 mL/min/1.73m^2^) had better eGFR improvement. In contrast to LdT, TDF, an acyclic nucleotide analogue structurally similar to adefovir, has been shown to be nephrotoxic [16]. In HIV-infected patients, TDF therapy has been associated with a modest decline in serum creatinine clearance [17]. Although there were no major changes in renal function in TDF-naïve CHB patients in clinical trials and real-world studies [18, 19], a careful screening for pre-existing renal risk factors and a close monitoring of serum creatinine and eGFR, phosphatemia, proteinuria, glycosuria and phosphaturia are mandatory for starting and continuing the therapy with TDF, especially in mild renal impairment in individuals with pre-existing risk factors for renal disease [20]. Among these studies, however, there were still many limitations, such as the lack of detailed drug history except NAs, the severity of diabetes or hypertension, and the eGFR status prior to NAs treatment, which are potential confounding risk factors for CKD. In this study, we identified CHB patients with impaired renal function (30–90 mL/min/1.73m^2^), no prior history of diabetes and hypertension, and no chemotherapy at enrollment in order to decrease the confounding factors in renal function. Finally, we confirmed that LdT and ETV are associated with significant improvement in eGFR, and TDF is associated with a decrease after a 2-year treatment. Of these groups, the stable renal function prior to NAs treatment was noticed by serial eGFR follow-up. Different from previously published studies, this is the first study comparing eGFR from prior to NAs treatment to after two years of treatment.

It is worth noting that ETV increases the eGFR after a 2-year treatment in the status of pre-existing renal impairment, despite no significant difference in CKD stage change. To the best of our knowledge, this is the first study indicating that ETV has a renal protective effect in CHB patients with renal impairment and without a history of diabetes and hypertension. Even though our results indicate that ETV improves renal function, many retrospective studies showed no improvement in eGFR after ETV treatment [19, 21, 22]. However, these studies did not exclude diabetic and hypertensive patients, who carry risk factors for renal function deterioration. A large cohort study from Turkey showed that ETV did not change eGFR from baseline to after a 2-year treatment (96.2 ± 22.5 → 95.9 ± 23.9 ml/min/1.73m^2^) [21]. However, there were 8.9% and 10.3% of patients with diabetes and hypertension, respectively, and up to 20% ETV-treated patients shifted from 60–90 ml/min/1.73m^2^ to > 90 ml/min/1.73m^2^. Therefore, a portion of patients with renal function improvement after ETV treatment indeed exists. The underlying mechanism needs further evaluation.

Despite the significant change in eGFR after NAs treatment, critics might argue the inconsistency in laboratory measurement of serum creatinine concentration, and day-to-day physiological variability in true GFR [23]. Small fluctuations in GFR are common and might not necessarily indicate progression. The greater the fluctuation in kidney function, the higher the probability of nonlinear progression. Hence, some studies also analyzed renal function by the change of eGFR category (i.e. from eGFR 60–90 to >90 mL/min/1.73m^2^) [4, 8]; however, cases with small changes in eGFR (for example, from 59 to 61 mL/min/1.73m^2^) would be inappropriately represented in an improvement group. An approach involving an assessment of change in eGFR category confirmed by a minimal percentage of change in eGFR (25% or greater) was recommended to define progression [24]. In the present study, only 4 patients (2.8%) are defined as a certain rise (change in eGFR category with >25% eGFR increase from baseline), and are all from the LdT group. It is noteworthy that there were 4 patients (2.8%) defined as a certain drop (change eGFR category with >25% eGFR decrease from baseline); and of these patients, 3 (75%) are from the LdT group. To sum up, LdT-naïve CHB patients revealed a higher proportion of eGFR fluctuations. We presumed the higher fluctuation might be related to the polymyopathy caused by LdT, although symptoms of muscle pain were rarely recorded in medical charts and most patients lack creatine phosphokinase measurements. We believed polymyopathy was under-diagnosed. However, it is still difficult to draw a conclusion by the definition of eGFR category change plus an eGFR difference of more than 25% in the present study because of limited patient numbers. A further large-scale prospective study is needed to clarify this.

The following question remains what outcomes result from these NAs-naïve CHB patients with a change of eGFR. Many population-based studies have reported the associations between a change in kidney function over time and adverse outcomes [25–27]. Several possible mechanisms for the observed association of a decline in kidney function and an increase in mortality are mentioned, including aggravation cardiovascular risk factors, endothelial dysfunction, oxidative stress, or vascular damage. It is noteworthy that the study of Turin *et al*. [28] described an increase mortality in subjects whose eGFR either increased or decreased over a 3-year period. The authors speculated the increased mortality, in those with an increased eGFR, might be due to either recovery from an episode of acute kidney injury, or lower serum creatinine generation as a result of reduced muscle mass from an associated mortal illness. In our study, there were only 1, 1, 4, 0, 1 mortality cases in certain rise, uncertain rise, stable, uncertain drop, and certain drop groups, respectively. All of these patients passed away due to liver cirrhosis complications, such as HCC, spontaneous bacterial peritonitis, or esophageal varices bleeding, and not from cardiovascular events, the major etiology of mortality in Turin’s study [28]. Therefore, in our analysis, although TDF resulted in a significant decrease after a 2-year treatment, there was no significant change in CKD stage compared to LdT and ETV, and no increased risk in mortality or renal complications. This result is similar to a recent large cohort study from Hong Kong [29], in which they used a total of 53,500 CHB patients (46,454 untreated and 7,046 treated with NAs) to evaluate the relative risk of renal (incident renal failure and renal replacement therapy) and bone (incident hip, vertebral, and all fractures) events. Finally they concluded that NA treatment does not increase the risk of renal and bone complications in CHB patients.

It is interesting to note that the high prevalence (33%, 218/647) of renal impairment in CHB patients without a history of DM, hypertension and chemotherapy in the present study. As we know, diabetes (43.2%) and hypertension (8.3%) are two major risk factors for developing CKD [30]. However, a close relationship exists between CHB and CKD, which may be of multiple origins. Epidemiological studies have shown that CHB in some individuals may lead to renal dysfunction through immune complex-mediated glomerular diseases, such as membranous nephropathy [31]. A large cohort study from France indicated that renal abnormalities are highly prevalent (64.6%) in CHB patients, in which diabetes and hypertension were observed in 4.6% and 9.2% of patients, respectively [32]. A cross-sectional study from southern Taiwan indicated that there was no significant association between proteinuria and HBV infection, but the prevalence of proteinuria among CHB was 6.4% [33]. From our data and prior epidemiological studies, clinical physicians must be conscious of monitoring renal function before and after treatment with potentially nephrotoxic agents.

In summary, in NAs-naïve CHB patients with impaired renal function, LdT and ETV resulted in a significant increase in eGFR while TDF resulted in a significant decrease after a 2-year treatment. Interesting, TDF had the smaller proportion of patients, but LdT had a higher proportion reclassified to a new category of CKD confirmed by a minimal percent change in eGFR (25% or greater). Whether NAs treatment affects the outcomes in those with a change in eGFR, especially in the population with an increase, has yet to be determined.

## Supporting Information

S1 Dataset General data of the study population. (XLS)
